# Detection of Aβ plaque-associated astrogliosis in Alzheimer's disease brain by spectroscopic imaging and immunohistochemistry†
†Electronic supplementary information (ESI) available. See DOI: 10.1039/c7an01747b


**DOI:** 10.1039/c7an01747b

**Published:** 2017-12-04

**Authors:** Francesca Palombo, Francesco Tamagnini, J. Charles G. Jeynes, Sara Mattana, Imogen Swift, Jayakrupakar Nallala, Jane Hancock, Jonathan T. Brown, Andrew D. Randall, Nick Stone

**Affiliations:** a University of Exeter , School of Physics and Astronomy , Exeter EX4 4QL , UK . Email: f.palombo@exeter.ac.uk; b University of Exeter , Medical School , Hatherly Laboratories , Exeter EX4 4PS , UK; c University of Exeter , Centre for Biomedical Modelling and Analysis , Exeter EX2 5DW , UK; d University of Perugia , Department of Physics and Geology , Perugia I-06100 , Italy; e University of Reading , School of Pharmacy , Reading Hopkins building , Reading RG6 6UB , UK

## Abstract

Recent work using micro-Fourier transform infrared (μFTIR) imaging has revealed that a lipid-rich layer surrounds many plaques in post-mortem Alzheimer's brain. However, the origin of this lipid layer is not known, nor is its role in the pathogenesis of Alzheimer's disease (AD). Here, we studied the biochemistry of plaques *in situ* using a model of AD. We combined FTIR, Raman and immunofluorescence images, showing that astrocyte processes co-localise with the lipid ring surrounding many plaques. We used μFTIR imaging to rapidly measure chemical signatures of plaques over large fields of view, and selected plaques for higher resolution analysis with Raman microscopy. Raman maps showed similar lipid rings and dense protein cores as in FTIR images, but also revealed cell bodies. We confirmed the presence of plaques using amylo-glo staining, and detected astrocytes using immunohistochemistry, revealing astrocyte co-localisation with lipid rings. This work is important because it correlates biochemical changes surrounding the plaque with the biological process of astrogliosis.

## Abstract 2

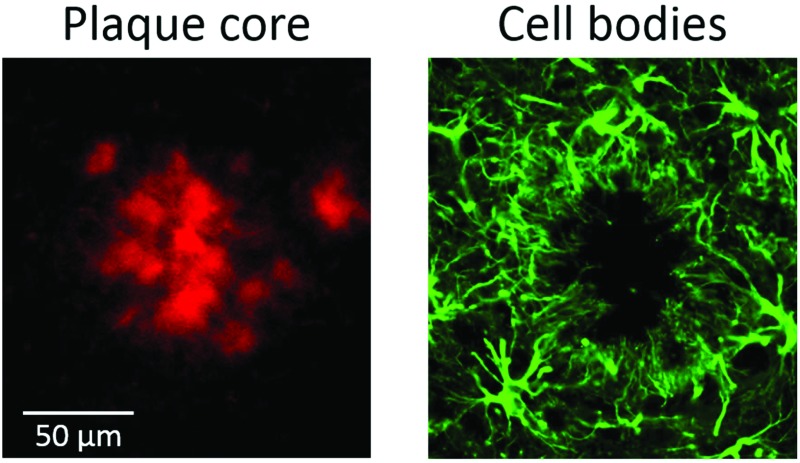
Correlative vibrational spectroscopy and immunohistochemistry reveal astroglial processes co-localised with the lipid-rich shell of Aβ plaques.

## Introduction

Alzheimer's disease (AD) is a progressive neurodegenerative disease and the most common form of dementia worldwide. There are currently 47 million affected people, a number that is predicted to double every 20 years,^1^ hence posing a significant burden in healthcare management of an ageing population. Amyloidopathy is a major hallmark of AD and consists in the abnormal accumulation in the brain parenchyma of the amyloid-β peptide (Aβ), an aggregation-prone protein fragment of 40–42 amino acids produced by the cleavage of amyloid precursor protein (APP). The monomeric form of the Aβ peptide is thought to physiologically exist as a soluble molecule in the brain; in some cases, Aβ monomers can progressively aggregate forming oligomers until they eventually precipitate into insoluble fibrils and finally nucleate to form insoluble plaques.^2^,^3^ Soluble oligomers have increasingly being recognised as the neurotoxic form of Aβ that can also be found, as a marker of the disease, in blood and cerebrospinal fluid.^4^–^6^ The Aβ peptide is the main but not the only component of amyloid plaques. The oligomeric composition of the deposits determines the biological effect and plausibly the clinical outcome of β-amyloidosis.^7^ Neurotoxicity seems to arise from early accumulation of soluble oligomeric species, rather than insoluble fibrils (with prevalent antiparallel β-strands^8^) or larger plaque formations.

The lack of a diagnostic tool for AD *in vivo* has prompted early attempts of applying vibrational spectroscopy techniques to investigate the molecularity and structure of Aβ plaques in the brain of both humans and mouse models of AD.^9^ Intriguingly, these studies have revealed that many plaques contain a lipid-rich layer surrounding the plaque. Dense core plaques in a (TgCRND8) transgenic mouse brain are composed of intermolecular β-sheet structures of the Aβ peptide, with significant increase in lipids around the core.^10^ Though the origin of this lipid layer has been the focus of several studies, including some based on multiphoton microscopy, a definitive hypothesis and mechanism of whole plaque formation is still lacking.

Significant advances in μFTIR spectroscopic imaging of AD brain have been brought about by Gough and co-workers, showing that it is possible to achieve enhanced spatial resolution in focal plane array (FPA) detection of plaques using synchrotron light source^10^–^12^ or high magnification optics.^13^ μFTIR imaging has also been applied to probe early conformational changes of Aβ and APP in AD mouse brain (Tg19959) that precede plaque formation and localize to synaptic terminals.^14^ Previous work using Raman microscopy^9^,^15^ has focused on the detection and chemical characterization of Aβ deposits, but there is still a lack of a combined use of Raman and FTIR imaging in order to obtain detailed information about the structure and chemical composition of plaques within the brain.

An in-depth investigation of the molecular species involved in the mechanism of Aβ plaque formation is the first step to unravel the pathophysiology of this complex disease. The aim of this work was to investigate the chemical and structural character associated with the distribution of specific compounds within amyloid plaques. Although some hypotheses have previously been made, no definitive conclusion around the origin of the lipid-rich ring surrounding the plaque core has yet been made. We clarified this for the first time in the present work using chemical imaging and correlative immunofluorescence imaging of plaques.

In this work, we used a TASTPM transgenic mouse model of AD characterized by Aβ overexpression. TASTPM mice carry a double mutation on the APP and one single mutation on the presenilin-1 gene resulting in severe accumulation of amyloid plaques and cognitive impairment by the sixth month of age.^16^,^17^ Using fixed brain histological sections obtained from TASTPM mice, we analysed the Aβ deposits within the hippocampus, a brain area that is involved in memory encoding and is severely affected in AD. Here, we provide evidence that plaque-surrounding astrocytic processes correlate with the presence of elevated lipid signals in the region surrounding the plaque core.

## Results

### μFTIR and immunofluorescence

**Fig. 1:**
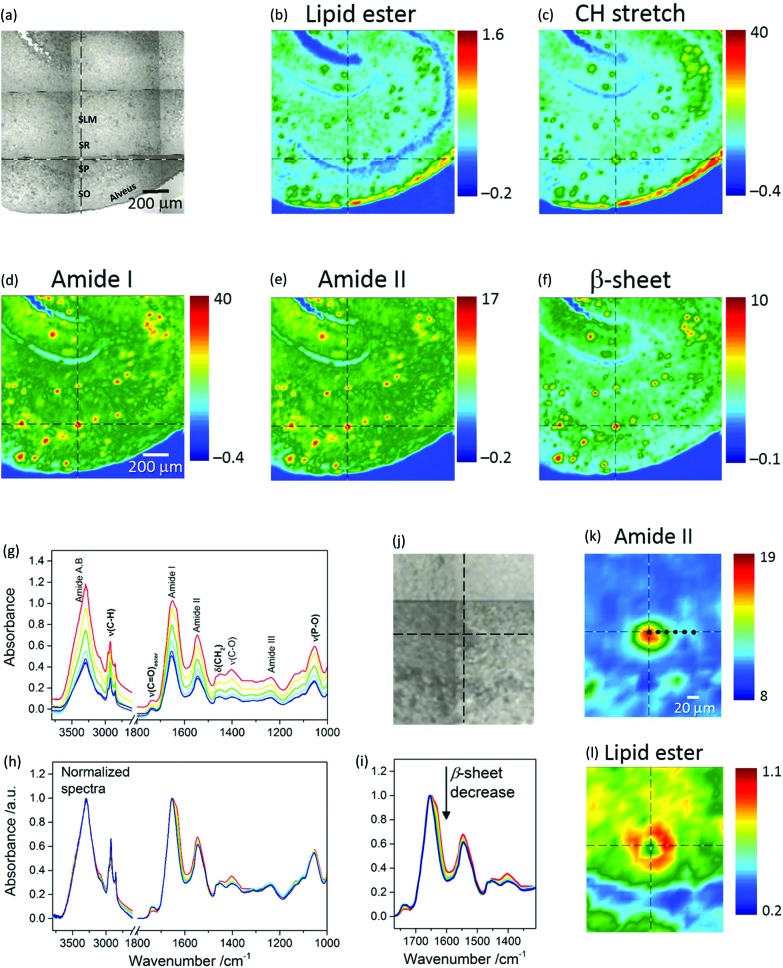
(Top panel) Visible and μFTIR images of a section of transgenic mouse brain containing the hippocampus. (a) Photomicrograph. Acronyms, which identify specific regions of the hippocampal CA1 region, are defined in the text. The FTIR images refer to the distribution of the integrated absorbance of (b) lipid *ν*(C=O)_ester_ (1761–1722 cm^–1^), (c) *ν*(CH) (3003–2805 cm^–1^), (d) amide I (1716–1595 cm^–1^), (e) amide II (1593–1483 cm^–1^), and (f) intermolecular β-sheet structure (1640–1620 cm^–1^ with baseline at 1716–1595 cm^–1^). (Bottom panel) Spectra, visible and μFTIR images of an amyloid plaque extracted from the same measurement. The FTIR images refer to the distribution of the integrated absorbance of (k) amide II and (l) lipid *ν*(C=O)_ester_ bands. The lipid ring can be clearly seen in (l). Cursors indicate the same pixel in correspondence of the plaque core. Black dots in (k) indicate the pixels from which FTIR spectra were selected along a horizontal line from the core through to the margin of the plaque. Spectra are colour coded to match the colours in image (k). (g) Main absorption bands are assigned to NH stretching (amide A and B; 3600–3000 cm^–1^), CH stretching (3000–2800 cm^–1^), lipid *ν*(C=O)_ester_ (1761–1722 cm^–1^), amide I (1718–1600 cm^–1^), amide II (1590–1480 cm^–1^), phosphate and amide III (1270–1180 cm^–1^), phosphate and sugars (1096–1016 cm^–1^). Bold labels refer essentially to lipid bands. (h) FTIR spectra normalized to the maximum of NH stretching and amide I bands for the regions at high and low wavenumbers, respectively. Note that no baseline correction was applied to the spectra. (i) Arrow indicates the spectral change, *i.e.* a decrease in intermolecular β-sheet structures, going from the core to the periphery of the plaque, corresponding to the black dots in (k).

Fig. 1a shows a photomicrograph of a transgenic (TG) mouse brain section, whereby the CA1 subfield of the hippocampus is easily recognizable because of the shell-shaped curvature of the tissue. Amyloid plaques can be visually identified as dark spots under white light due to light scattering.

Micro-transmission FTIR images from this location are presented in Fig. 1b–f. Each chemical image represents a plot of the integrated absorbance of a specific band in the IR spectrum across the area imaged by the FPA detector.

Protein distribution images (Fig. 1d–f) show the prevalence of Aβ plaques identified as red spots in this region. Red in the pseudo-colour images corresponds to high absorbance hence high concentration of the molecular species, whilst blue corresponds to low absorbance hence low concentration. Fig. 1f is an image based on the amide I sub-peak at 1628 cm^–1^, which is distinctive for intermolecular β-sheet structures.^18^ Images of the lipids (Fig. 1b and c), based on the ester carbonyl stretching (*ν*(C=O)_ester_) at 1735 cm^–1^ and CH stretching (*ν*(CH)) in the range 3003–2805 cm^–1^ obtained from the same measurement, show lipids as green regions mostly co-localized with the plaques. The blue arc in Fig. 1b denotes the *Stratum Pyramidale* (SP), which is the cell body layer, where lipids are less abundant. *Stratum Oriens* (SO), *Stratum Radiatum* (SR) and *Stratum Lacunosum Moleculare* (SLM), all containing non-myelinated components such as axonic and dendritic processes (from CA1 pyramidal neurons, apical and basal dendrites, and other CA1 inter-neuronal processes) and sparse cell bodies, have intermediate lipid content and are shown as cyan. The red arc in Fig. 1b and c identifies a layer that corresponds to the alveus, containing myelinated axons (myelin electrically insulates axons and speeds neural conduction) projecting from the CA1 subfield of the hippocampus to other brain areas.

The distinctive structure of Aβ deposits is clearly visualized in Fig. 1k and l, which present the dense core plaque identified by the crosshair cursor in Fig. 1j. The distributions of the protein and lipid ester bands show a dense core that is rich in polypeptides (Fig. 1k) and a ring-shaped region around the core rich in lipids (Fig. 1l). Spectra selected from different regions of the plaque show (Fig. 1g) a gradient of absorbance, which decreases from the core through to the periphery due to a decrease in thickness of the tissue section and/or in tissue density. The corresponding normalized spectra (Fig. 1h) show relevant differences between profiles: the plaque core spectrum (red line) has a doublet bandshape for the amide I mode, with a maximum at 1651 cm^–1^, generally assigned to α-helix, and a shoulder at 1628 cm^–1^, associated with β-sheet;^10^ conversely, the periphery of the plaque has a single broad band at 1654 cm^–1^. The observed spectral change indicates a gradual decrease of protein signals (Fig. 1i) due to the intermolecular β-sheets (at 1628 cm^–1^), amide II and the amyloid band (plausibly due to CH deformation, at 1404 cm^–1^ (ref. 12)) accompanied by an increase of lipid bands (CH stretching at 2923 and 2852 cm^–1^, which are due to the asymmetric and symmetric stretching vibrations of lipid acyl CH_2_ groups, respectively; *ν*(C=O)_ester_ at 1738 cm^–1^) when going from the core through to the periphery of the plaque. To analyse these variations, difference spectra were calculated (see below). Fig. 1 shows representative images of FTIR-visualised plaques – more samples from different mice and more sections from the same mice can be found in the ESI (Fig. SI-1†).

**Fig. 2:**
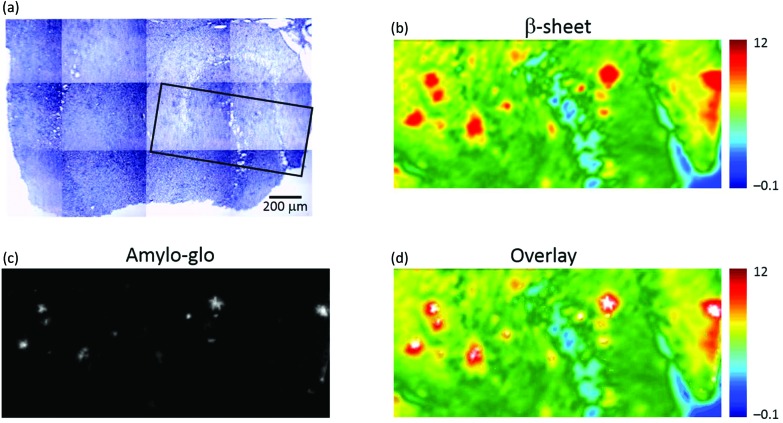
Visible, μFTIR and fluorescence images of a section of transgenic mouse brain containing the hippocampus. (a) Photomicrograph; dense core plaques appear darker than surrounding tissue. The box (black) indicates an area where a fluorescence image was obtained. (b) FTIR image showing the distribution of the integrated absorbance of the intermolecular β-sheet structures in the range 1645–1622 cm^–1^ (baseline at 1716–1595 cm^–1^). (c) Fluorescence image of the section stained with amylo-glo for Aβ peptide. (d) Composite image formed by overlaying the FTIR and fluorescence (white pixels) images.

To confirm that the plaques identified with μFTIR imaging were indeed genuine plaques, we compared the FTIR images directly with immunofluorescence imaging based on amylo-glo staining of the same specimen (see Fig. 2). Amylo-glo fluorescence assay is a consolidated “gold standard” for the detection and quantification of Aβ.^19^Fig. 2d shows an overlay between the FTIR image (rainbow) and the amylo-glo staining (white).

The hippocampi of wild type (WT) mice entirely lack plaque pathology. μFTIR imaging applied to *ex vivo* hippocampal sections from WT mice (Fig. SI-2†) reveal no occurrence of the plaque structures observed in TG animals. The distributions of intermolecular β-sheet (Fig. SI-2a and d†) do not present the focal accumulations representative of plaques observed in the TG mice brain (see *e.g.*Fig. 1f). Similarly, the lipid distributions (Fig. SI-2b and e†) show no appearance of ring-shaped regions, but instead simply outline the normal layered anatomical structure of the hippocampus. Staining with the amyloid-specific probe amylo-glo did not reveal the presence of any plaques in these samples (Fig. SI-2c and f†).

### Correlative FTIR imaging and Raman mapping

Combining Raman and infrared imaging is a powerful tool as FTIR can rapidly scan large fields of view so that images of plaques can be made, while Raman microscopy has higher spatial resolution (diffraction limited to *λ*/2NA = 0.8 μm) but, in general, is a much slower technique restricting the number of plaques that can be analysed. To our knowledge, this is the first time that a side-by-side comparison of Raman with μFTIR has been performed on individual plaques.

**Fig. 3:**
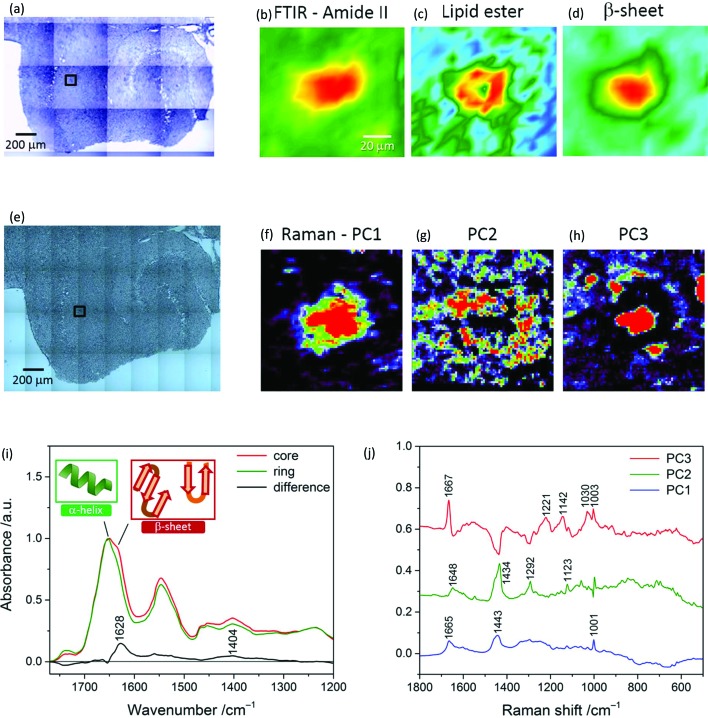
(Top and middle panels) Photomicrographs, μFTIR images and Raman principal component analysis (PCA) score maps of a transgenic mouse brain hippocampal section. Black box in (a) indicates a specific plaque area within the whole tissue FTIR image. The FTIR images refer to the distribution of the integrated absorbance of the (b) amide II (1588–1482 cm^–1^) and (c) lipid *ν*(C=O)_ester_ band (1761–1722 cm^–1^), and of the (d) height of the β-sheet peak (at 1628 cm^–1^). Colour scale limits are (b) 15 to –1.5, (c) 1.5 to 0.5, and (d) 0.9 to –0.09. Black box in (e) denotes a 99 × 99 μm^2^ area (same plaque as in a) where a Raman map was acquired using a 1.4 μm step-size. Map scores were derived from PCA applied to the Raman map and refer to the distribution of (f) average spectrum, (g) lipid-rich envelope and (h) β-sheet protein core(s) of the plaque. (Bottom panel) Representative FTIR spectra and Raman loading plots of a plaque. (i) Spectra extracted from a μFTIR image (Fig. 1k) and normalized with respect to the amide I band peak absorbance. Amide I peak position is 1651 cm^–1^ for the core and 1654 cm^–1^ for the ring, indicative of protein's α-helix conformation; the core spectrum has a doublet bandshape for the amide I mode. A difference spectrum was obtained by subtracting the ring profile from the core profile; it shows a loss of absorbance for the core spectrum at *ca.* 1738 cm^–1^ (lipid esters) and a gain at 1628 cm^–1^ (intermolecular β-sheets) and 1404 cm^–1^ (not fully clarified signature of the plaque core, slightly shifted to the position found in a different mouse model^12^). (j) Loading plots extracted from PCA applied to the Raman map (in the middle panel). PC3 corresponds to the core spectrum and presents the distinctive amide I symmetric peak of the β-sheet conformation at 1667 cm^–1^,^15^ whilst PC2 represents the ring, with resonances due to lipids (distinctive bands at 1434 (CH_2_ deformation) and 1123 cm^–1^ (C–C stretching)) and other protein conformations (1648 cm^–1^, assigned to α-helix and random coils).^20^ PC1 denotes the mean spectrum of the tissue.

Correlative μFTIR imaging, Raman mapping and fluorescence imaging were conducted on TG and WT mice brain specimens. Raman microscopy revealed the presence of β-pleated sheet conformation in the plaque core and the lipid ring around it, as shown in Fig. 3.

Fig. 3a and e display a TG hippocampal section visualised under the FTIR microscope (15× objective) and Raman microscope (20×), respectively; the latter photomicrograph shows the area where a Raman map of a plaque in CA1 was acquired. Fig. 3b–d are portions of FTIR mosaic images of a plaque based on the protein (amide II), lipid ester and β-sheet peak (height) distribution, respectively. PCA was applied to the Raman map (72 × 72 points at 1.4 μm distance) and the first three components were analysed. PC1, which represents the average spectrum of the sample, is shown in Fig. 3f, whilst Fig. 3g and h are score maps of PC2 and PC3, respectively, which give the distribution of the lipid ring surrounding the plaque core and the β-pleated sheet protein conformation – mainly concentrated in the core. From these images it is apparent that the higher spatial resolution of Raman microscopy enables a detailed analysis of the plaque structure, as shown in Fig. 3f–h. Multivariate analysis applied to all Raman datasets aided in extracting the relevant information that otherwise was difficult to disentangle through a univariate spectral analysis.

**Fig. 4:**
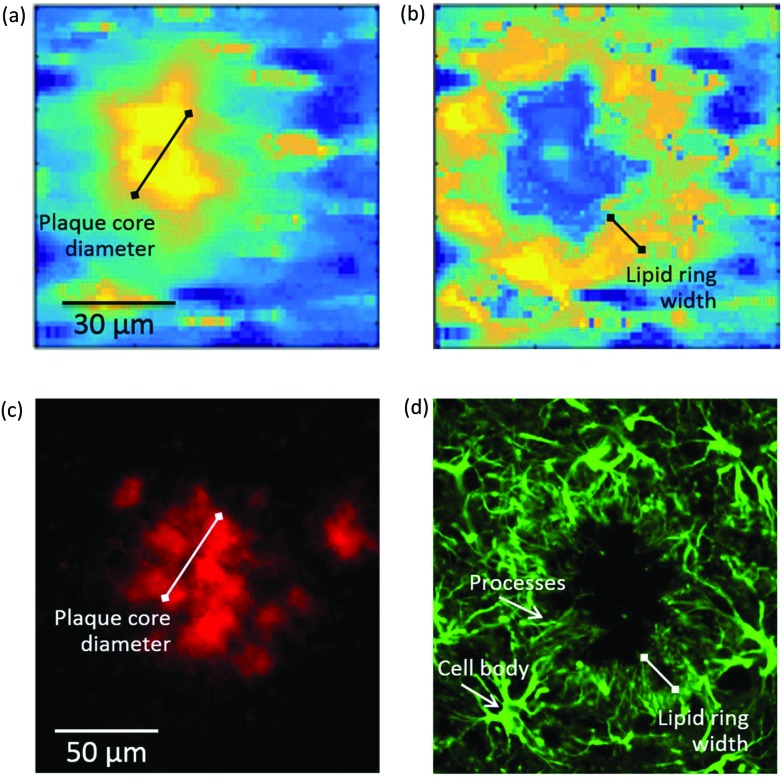
(Top panel) Self-organising map principal component analysis (SOM PCA) results derived from a Raman map of a plaque in a transgenic mouse brain hippocampal section. The Raman map was acquired over a 92 × 92 μm^2^ area using a 1.4 μm step-size. Map scores refer to the distribution of the (a) SOM PC1, which denotes the β-sheet core of the plaque, and (b) SOM PC2, showing the lipid halo surrounding the plaque core. Corresponding loading plots are shown in Figure SI-3.† (Bottom panel) Immunofluorescence images at 20× magnification of a large plaque within a section of TG mice brain containing the hippocampus stained with (c) amylo-glo for Aβ peptide and (d) GFAP for astroglia. The plaque represented in (a) and (b) is different from the one in (c) and (d). Arrows indicate the location of processes and cell bodies. Segments denote the size of the plaque core and lipid ring, providing a measure of the lipid ring width-to-plaque core diameter of approximately 0.5 in both types of images.

These results show that (1) Raman can reveal the same structures as can be seen with FTIR imaging (*i.e.* protein rich core and lipid ring), whilst also showing additional features such as cell bodies (Fig. 4b – more discussion on this follows); (2) Raman has better resolution than FTIR and can reveal similar chemical structures; it is also much slower and cannot be easily applied to a large field of view, which is an advantage of using FTIR.

Fig. 3i illustrates representative μFTIR spectra of a plaque in a TG mouse brain sample. The spectrum of the core presents the weakest absorbance of the lipid band (C=O stretching at 1738 cm^–1^), indicating a lower lipid concentration in the core than in the ring around the plaque, in accordance with previous observations.^21^ A change in bandshape of the amide I band is clearly revealed here, the core spectrum showing a maximum at 1651 cm^–1^, which is shifted at 1654 cm^–1^ in the ring spectrum. This spectral effect may be attributed to a change in the secondary structure of the proteins in the two sub-regions of the plaque. At these high frequencies, absorption due to the α-helix conformation is expected;^10^ a lower wavenumber position of the amide I band maximum in the core profile correlates with an increase of (β-sheet) ordered structure for the proteins in the plaque core area, while less ordered peptide structures would characterize the ring. Such an effect has been previously reported for both the Aβ peptide^21^ and other proteins.^22^,^23^ Here the enhancement of absorbance at 1628 cm^–1^ (ordered conformation), highlighted by the difference spectrum in Fig. 3i, can be referred to the peptide's intermolecular β-sheet structures which are characteristic of the core.

Fig. 3j is a plot of the three PCs’ loadings derived from PCA applied to the Raman map of a plaque (Fig. 3f–h). The plots are labelled based on the structures they represent: PC1-mean spectrum of the tissue, PC2-ring, and PC3-core. The main resonances in these plots are also reported. Notable differences characterize the amide I region, with the core exhibiting a symmetric intense peak at 1667 cm^–1^ due to β-pleated sheet,^15^ and the ring showing signatures of lipids and other protein conformations, *e.g.* α-helix.^20^

### Cellular and functional correlates

To elucidate the origin of the lipid ring around the amyloid plaque core detected by FTIR imaging and Raman microscopy, we performed a second subset of experiments using amylo-glo and immunofluorescent co-labelling with antibodies for astroglia (GFAP). These cell types have previously been reported to cluster around plaques.^24^–^28^

To establish if there was a link between the astrocyte processes and the lipid ring, we compared the ratio of the width of the lipid ring to the diameter of the plaque core for a number of plaques in both the immunofluorescent, μFTIR and Raman images. An example of this measurement can be seen in Fig. 4, with a Raman image of a plaque alongside an immunofluorescence image. Using the ratio of lipid ring to plaque core size provided a robust relative measure that could be used on a number of differently sized plaques, in different samples. We obtained a ratio of 0.45 ± 0.05 (*N* = 30 plaques) for all plaques visualised by μFTIR, Raman and immunofluorescence. As the lipid ring has approximately the same width as the astrocyte processes, and they co-localise in the same position around the plaque core, we conclude that this is compelling evidence that the lipid ring is associated with the astrocyte processes.

More examples of immunostained plaques can be seen (Fig. SI-4†) which reveal that astrocytes cluster around the vast majority of the plaques and have cell bodies located in the outer rim of the cluster while radially projecting processes (*i.e.* membrane extensions) towards the plaque core. This is a feature of astrogliosis in the brain parenchyma.

Interestingly, we also show how Raman has the spatial resolution to detect cell bodies. In Fig. SI-3c,† the Raman map shows cell bodies in the region surrounding the lipid-rich halo of the plaque that are revealed as small ovoids of *ca.* 10 μm diameter (yellow in the pseudo-colour map). The presence of nearby cell bodies has previously been revealed by synchrotron-based μFTIR imaging but not by globar source μFTIR,^12^ suggesting that high spatial resolution is a requirement for (unlabelled) detection of these small features. The Raman maps presented here give high definition for the cell bodies and comparable spatial resolution to the synchrotron FTIR images. Similar cell bodies can also be seen in immunofluorescent images (Fig. 4d).

## Discussion

The chemical structure and formation of amyloid plaques is a much studied, but still controversial topic. Here we have used spectroscopic tools to chemically analyse plaques *in situ*, in combination with conventional fluorescence stains to visualise plaques and surrounding biological processes. Our results are consistent with the hypothesis that a lipid ring, which is seen surrounding many plaques, is associated with astrocyte processes. This is evidenced by immunohistochemistry images that show astrocyte processes surrounding plaques (stained with amylo-glo) and co-localising with the lipid ring.

μFTIR imaging and Raman micro-spectroscopy were applied to study the chemical composition of Aβ plaques in the hippocampal CA1 region of the brain of TASTPM mice, an Aβ-overexpressing transgenic mouse line. Previous studies have used μFTIR imaging for the visualization of amyloid plaques and have found lipid rings surrounding some plaques.^21^,^29^ However, this is the first time that both FTIR imaging and Raman microscopy were applied through a site-matched approach in order to fully describe the structure and composition of these plaques. We observed changes in plaque composition between the core and the periphery (ring, or shell if considered in 3-D). The core appeared to be richer in ordered (intermolecular β-pleated sheet) structures, the concentration of which tends to progressively decrease from the core through to the periphery of the plaque; on the other hand, lipids appeared to be less concentrated in the core of the plaque, instead forming a ring-shaped distribution co-localized with less ordered protein structures. The progressive reduction of β-pleated sheet structures from the core to the periphery of the plaque was confirmed through Raman maps (with NIR 830 nm excitation).

Fluorescent labelling for Aβ peptide and astrocytes confirmed that some plaques are clustered with astrocytes; this is paralleled by the observation that some plaques have a lipid-rich layer around the core (detected by FTIR imaging and Raman mapping). Raman maps elucidate the finer details of the plaque structure, revealing small ovoids in the outer rim of the plaque that correspond to the cell bodies of astrocytes. Astrocytic processes radially extend from the cell bodies towards the core of the plaque, co-localizing with the lipid-loaded ring. As for equal volumes the surface/volume ratio (approximately corresponding to the lipid/water ratio in cells) is higher in elongated processes compared to ovoids, we infer that the astrocytic processes surrounding the plaques likely correspond to the origin of the lipid signal detected in this region. This is an important observation, as it reveals that the ring around the plaque can itself be a marker of astrogliosis. It should be highlighted that the lipid signal may also originate from the activated microglia surrounding Aβ plaques;^30^ however, astrocytes are more likely, as activated microglia are less ramified compared to non-activated microglia.^31^,^32^

As with microglia activation, astrogliosis is strongly associated with amyloidopathy. Several lines of evidence suggest that while the amyloid-dependent microglial activation is responsible for increased risk and more severe progression of AD,^30^,^33^ amyloid plaque-associated astrogliosis is negatively correlated to AD development.^24^ Post-mortem studies of the brain of people with Alzheimer's disease and non-demented age-matched controls have shown that amyloid plaques can also be found in the brain of non-demented controls, especially older people,^34^,^35^ suggesting that other factors might promote the pathogenesis and progression of the disease. Also, a recent study has shown that patients with lower mini mental state exam (MMSE) scores have a lower astrogliosis-associated plaque burden, suggesting that astrogliosis might have a protective role limiting the development of amyloidosis-dependent dementia.^24^

The ability of μFTIR imaging and Raman microscopy to detect both Aβ plaques and plaque-associated astrogliosis might allow the use of chemical imaging to identify astrogliosis and clustered inflammation in nerve tissue with amyloidosis, with potential for translation into an early diagnostic tool. The biochemical information this brings will likely prove invaluable for further elucidating the mechanisms of AD.

## Conclusions

In this paper, we have focussed on analysing the chemical composition of plaques in a transgenic mouse model of Alzheimer's disease, using μFTIR imaging and Raman microscopy, with correlative immunofluorescence imaging. We have found that the plaques tend to have a protein-rich core, high in intermolecular β-sheets, surrounded by a lipid ring. This ring correlates with processes from astrocytes (and possibly microglial cells), which we propose are the origin of the lipid-rich ring found in μFTIR images and Raman maps. This is a novel finding and adds weight to the hypothesis that plaques cause chronic inflammatory responses to the surrounding tissue, which could potentially be a root cause of neurodegeneration.

## Methods

### Animal procedures

Animal procedures complied with the UK Home Office Guidelines and the University of Exeter Animal Welfare Ethical Review Board. Male 12 months old Aβ-overexpressing TASTPM (TG) mice and age-matched littermate controls (WT) were used in this study. TASTPM mice carry two mutations on the gene for the amyloid precursor protein (APP_Swe_ K670N, M671L) and one on the presenilin 1 gene (M146 V) that can be found in patients affected by familial Alzheimer's disease.^16^,^17^ All animals were housed at room temperature under a 12 hours light cycle, and fed a normal diet with free access to food and water *ad libitum* before being sacrificed.^36^

### Tissue collection and sectioning

The animals were humanely sacrificed by cervical dislocation (Schedule 1, ASPA, 1986). The brain was rapidly removed and acute horizontal slices of 300 μm thickness were cut in a vibratome and suspended in artificial cerebrospinal fluid, as previously described.^36^ The major part of each brain was used for electrophysiological recordings. A number of slices of each brain (TASTPM and WT mice) containing ventral hippocampus, striatum and cortex were retained for the present study. The slices were post-fixed overnight with 4% formalin + 0.1 M phosphate buffer solution (PBS). They were rinsed twice (5 min each time) with 0.1 M PBS and stored in 30% (w/v) sucrose solution to inhibit subsequent formation of ice crystals, before being embedded in a water-soluble frozen section medium (NEG-50, Thermo Scientific) and snap frozen. Sections of 20 μm thickness were cut in a cryostat and left to rinse for at least 24 hours in 0.1 M PBS. Sections were subsequently rinsed in distilled water and mounted on Raman-grade polished calcium fluoride slides (Crystran, Poole, Dorset, UK).

### Microscopy and spectroscopy

Three brain sections per animal (five TASTPM and five WT mice) were analysed giving a total of 30 specimens. For two random sections, measurements were rerun to assess their repeatability.

Micro-transmission Fourier transform infrared (μFTIR) images were collected with an imaging system consisting of an Agilent Technologies Cary 670 FTIR spectrometer coupled to a Cary 620 FTIR microscope with a 0.62 NA, 15× Cassegrain objective, and a liquid nitrogen-cooled focal plane array (FPA) detector. The detector had 16 384 pixels arranged in a 128 × 128 array. The area imaged in μtransmission mode through the 15× objective corresponds to 704 × 704 μm^2^. Characteristic bands of proteins and lipids, principally amide I, amide II, ester carbonyl and CH stretching, were analysed, yielding images with a spatial resolution in the range of 5–10 μm (fingerprint region). These bands constitute the spectral signatures of the main components of amyloid plaques.^11^ Resolutions Pro v. 5.3 software was used for acquisition and manipulation of the data. An IR absorption spectrum was obtained for each detector pixel by co-adding 32 interferograms and applying a Fourier transform. The Blackmann–Harris 4-point apodization function was used. Spectra were measured over the range 4000–1000 cm^–1^ at a spectral resolution of 4 cm^–1^ with a zero filling factor of 2, achieving a spectral spacing of approximately 2 cm^–1^. Prior to all image measurements, a background obtained from a tissue-free region of the substrate was measured by co-adding 64 interferograms. Large images of the whole hippocampus were constructed as mosaics of individual tiles; each tile was selected to be 128 × 128 pixels.

FTIR imaging data are hyperspectral datasets, with two spatial dimensions and a third spectral dimension (wavenumber); each value in the datasets is absorbance of the sample at each combination of position and wavenumber. Absorbance is related to concentration of a chemical constituent through the Beer–Lambert law, thus enabling label-free compositional analysis. In the present work, the whole mid-infrared spectrum between 4000 and 1000 cm^–1^, containing the absorption bands of proteins and lipids, was measured for each detector pixel. To calculate the absorbance of each band, a linear baseline was drawn between the minima either side of the peak and absorbance values above this line were integrated with respect to wavenumber. This enabled chemical images to be obtained (univariate analysis).

Raman micro-spectroscopy maps were collected with a Renishaw inVia Raman microscope equipped with an 830 nm laser and a Leica long working distance 50× (NA 0.50) objective. The backscattered light from the sample was dispersed through a 600 gr mm^–1^ grating onto a Renishaw deep depletion CCD camera. The spectral resolution was approximately 7 cm^–1^. Raman maps were acquired in streamline mode with an exposure time of 55 s per laser line (equivalent to *ca.* 9 s per point) with step size of 1.4 μm, in the range 2400–450 cm^–1^. WiRE v. 4.0 software was used for acquisition and manipulation of the data. Accurate calibration of the set-up and focus adjustment of the objective was performed before each series of measurements. Spectra were collected with the focus position 8 μm into the specimen. Cosmic ray removal was applied to each spectrum using the nearest neighbour method. Spectral maps were then analysed by Principal Component Analysis (PCA) using 10 components and spectrum centring + normalization (SNV) pre-processing. In this manner, the first principal component (PC1) corresponds to the average spectrum of the sample. Results were reported as obtained, without any further modification including baseline correction.

Alternatively, when analysing the Raman maps using SOMs the cosmic rays were removed by the following method: cosmic rays were filtered from the Raman maps by applying a 3 × 3 window two-dimensional median filter to each wavenumber. For the 3 × 3 window used, the centre pixel for that wavenumber is replaced with the median value of all pixels in the window for that wavenumber. After the application of median filtering to each wavenumber, the collected Raman spectra were unfolded to create a large, two-dimensional data matrix where each row corresponds to the Raman spectrum of a single pixel and each column to a single wavenumber. At this stage the spectra were compressed using the Self Organising Maps (SOM) algorithm (as described in ref. 37), followed by extended multiplicative scatter correction, mean centring and then PCA.

The tissue samples analysed by μFTIR imaging and Raman microscopy were subsequently stained with amylo-glo reagent (Biosensis), mounted with fluoromount (Sigma) and imaged on a Nikon Eclipse EF-800 epifluorescence microscope. Staining was performed as previously reported;^19^ see also the ESI† for a detailed description of amylo-glo staining of Aβ plaques. To clarify the origin of the lipid signature in the region surrounding the plaque core in μFTIR images of TASTPM mouse hippocampus, slices were immunohistochemically stained for astroglia and co-stained with amylo-glo to check the pattern of distribution of astrocytes with respect to the Aβ plaque cores. See ESI† for a detailed description of the immunohistochemical procedures. All images were taken with an epifluorescence microscope.

## Author contributions

F.P. and F.T. conceived, designed and supervised the project. F.T. obtained and characterized the samples. J.C.G.J., J.H., S.M., I.S., F.P. and J.N. performed the experiments. F.P., F.T. and N.S. processed and analysed the data. J.T.B., A.D.R., J.H., J.N. and N.S. helped with the study design and discussion of the results. F.P. wrote the manuscript with input from all other authors.

## Conflicts of interest

The authors declare no competing financial interests.

## Supplementary Material

Supplementary information
