# Supplementary material for: Detection of Aβ plaque-associated astrogliosis in Alzheimer's disease brain by spectroscopic imaging and immunohistochemistry
Source: Analyst. 2017 Dec 4;143(4):850–7. doi: 10.1039/c7an01747b (PMC5851084; doi:10.1039/c7an01747b)
Supplement: Supplementary file 1 [file AN-143-C7AN01747B-s001.pdf]

Electronic Supplementary Material (ESI) for Analyst.
This journal is © The Royal Society of Chemistry 2017

Detection of Aβ plaque-associated astrogliosis in Alzheimer's disease brain by spectroscopic imaging and immunohistochemistry

Francesca Palombo[†]*, Francesco Tamagnini[‡$], J. Charles G. Jeynes[#], Sara Mattana[§], Imogen Swift[†], Jayakrupakar Nallala[†], Jane Hancock[‡], Jonathan T. Brown[‡], Andrew D. Randall[‡], and Nick Stone[†]

[†] University of Exeter, School of Physics and Astronomy, Exeter EX4 4QL, UK
[‡] University of Exeter, Medical School, Hatherly Laboratories, Exeter EX4 4PS, UK
[#] University of Exeter, Centre for Biomedical Modelling and Analysis, Exeter EX2 5DW, UK
[§] University of Perugia, Department of Physics and Geology, Perugia I-06100, Italy
[$] University of Reading, School of Pharmacy, Reading Hopkins building, Reading RG6 6UB, UK

Keywords: Alzheimer's disease; experimental model; amyloid fibrils; Aβ oligomers; vibrational spectroscopy, FTIR, Raman.

Immunostaining protocol

Mouse brain slices of 300 μm thickness were fixed in paraformaldehyde, subsequently incubated overnight in PBS 0.1% and sucrose 30% for cryoprotection and finally cryosectioned to 30 μm thickness using a Bright 7500 microtome (Bright Instruments). The sections were then incubated in blocking buffer for 1 hour (BSA 1%, NGS 3%, PBS) followed by treatment with staining agent anti-GFAP (1:200, Abcam) overnight at 4°C for astrocytic staining. Primary antibodies were visualized with Alexa Fluor® secondary antibodies (1:200, ThermoFisher Scientific). Sections were incubated in the secondary antibody for 4 hours at room temperature and then washed in PBS. Three 15-minute washes were performed between each step (0.1 M PBS, 0.3% triton X). For amyloid plaque staining, sections were incubated in 70% ethanol for 5 minutes, distilled water for 2 minutes and finally in amylo-glo RTD staining solution (Biosensis) for 10 minutes at room temperature. Slices were washed in 0.9% saline solution (NaCl) and mounted in Fluoromount (Sigma Aldrich). Immunofluorescence imaging was performed using a Nikon Eclipse EF-800 epifluorescence microscope. Images were processed using Image J software [1].

FTIR spectroscopic images

Transgenic mice

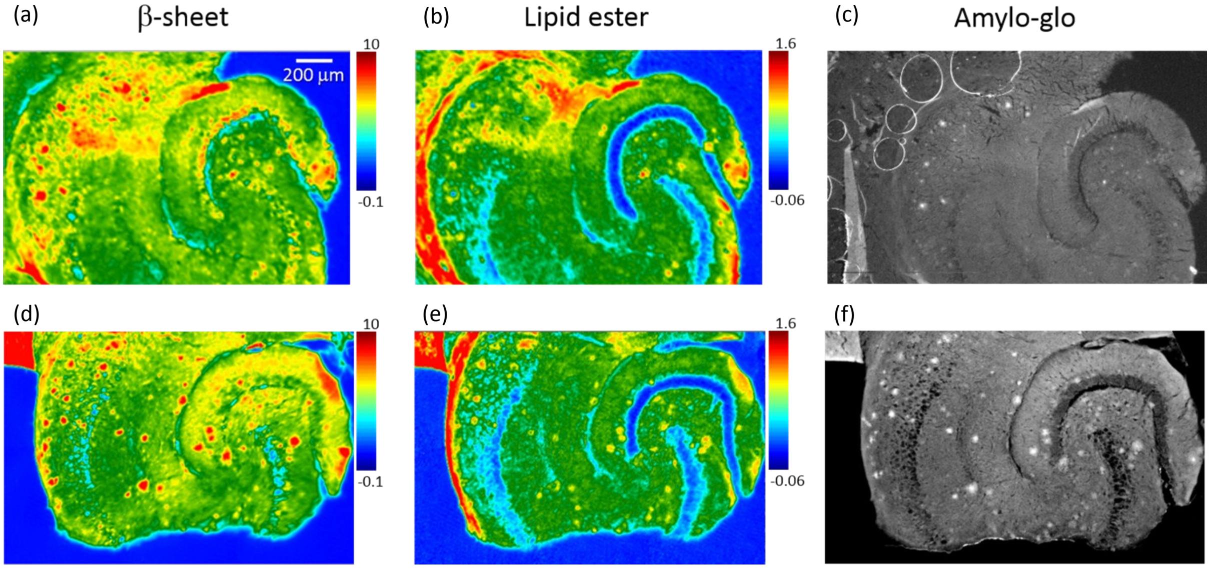

Figure SI-1. (<u>Top and bottom panels</u>) μFTIR and immunofluorescence images of two distinct sections of a TG mouse brain hippocampus. The FTIR images refer to the distribution of the integrated absorbance of **(a,d)** intermolecular β-sheet structures in the range 1645–1622 cm^{-1} (baseline at 1716–1595 cm^{-1}) and **(b,e)** lipid $\nu(C{=}O)_{ester}$ in the range 1761–1722 cm^{-1}. The same colour scale was selected, thus enabling a direct comparison of absorbance of the plaques and tissue between the images of the two sections. Blue regions denote the calcium fluoride substrate, i.e. absence of a tissue section. **(c,f)** Fluorescence image of the sections stained with amylo-glo for Aβ peptide, showing the presence of plaques.

Wild type mice

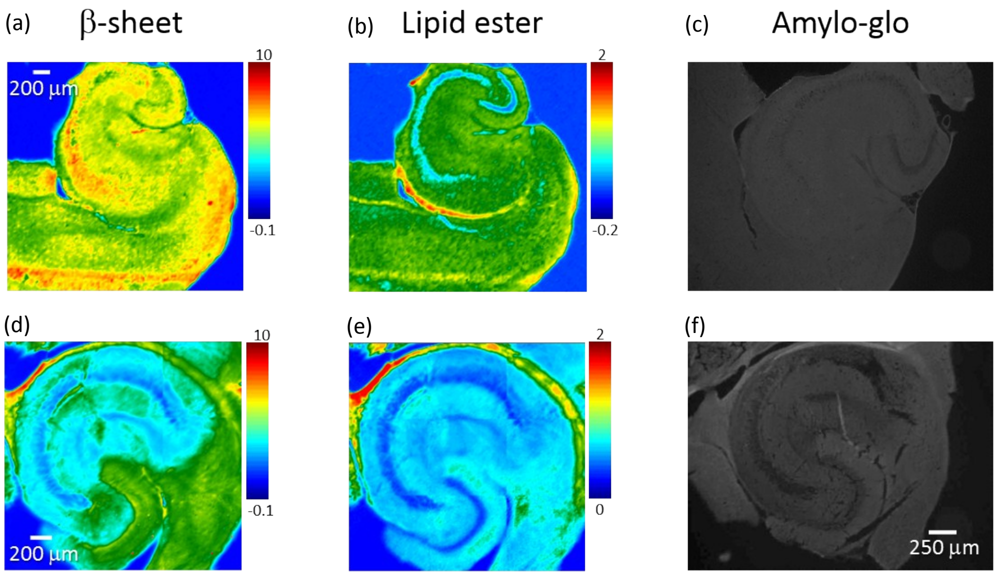

Figure SI-2. (<u>Top and bottom panels</u>) μFTIR and immunofluorescence images of two sections from two WT mice brain hippocampi. The FTIR images refer to the distribution of the integrated absorbance of the **(a,d)** intermolecular β-sheet structures in the range 1645–1622 cm^{-1} (baseline at 1716–1595 cm^{-1}) and **(b,e)** lipid $\nu(C{=}O)_{ester}$ band (1761–1722 cm^{-1}). **(c,f)** Staining with the amyloid plaque specific probe amylo-glo did not reveal the presence of any plaque in these samples.

Raman microspectroscopic maps

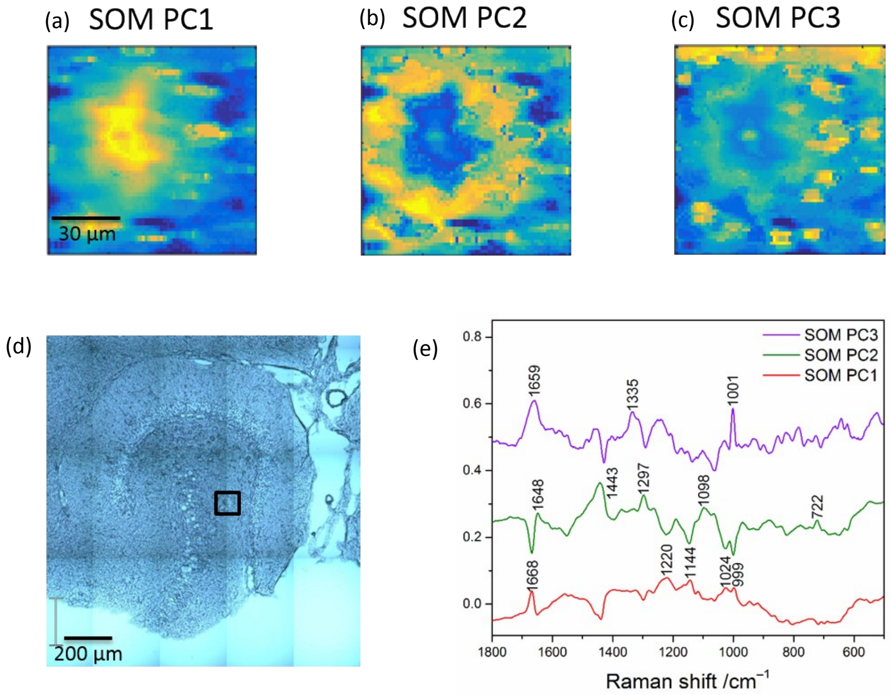

Figure SI-3. SOM PCA results derived from a Raman map of a plaque in a TG mouse brain hippocampal section. **(d)** Photomicrograph. The black box denotes a $99 \times 99\ \mu m^2$ area where a Raman map was acquired using a 1.4 μm step-size. (Top panel) Map scores refer to the distribution of **(a)** SOM PC1, which denotes the β-sheet core of the plaque, **(b)** SOM PC2, representing the lipid-rich halo around the plaque core, and **(c)** SOM PC3, showing cell bodies in the surroundings of the plaque. (Bottom panel) **(e)** Loading plots are assigned as follows: SOM PC1 (red line) corresponds to the plaque core spectrum and presents the distinctive amide I symmetric peak of the β-sheet conformation at 1668 cm^{-1}, whilst SOM PC2 (green line) represents the ring, with resonances due to lipids (distinctive bands at 1443 (CH_2 bending) and 1297 cm^{-1} (fatty acids[2]) and other protein conformations (1648 cm^{-1}, assigned to α-helix and random coils); SOM PC1 (violet line) shows bands due to proteins (1659 cm^{-1}, amide I vibration of α-helix, and 1001 cm^{-1}, phenylalanine) and nucleic acids (1335 cm^{-1}, CH_3CH_2 wagging) denoting the heterogeneous composition of cell bodies.

Immunofluorescence images

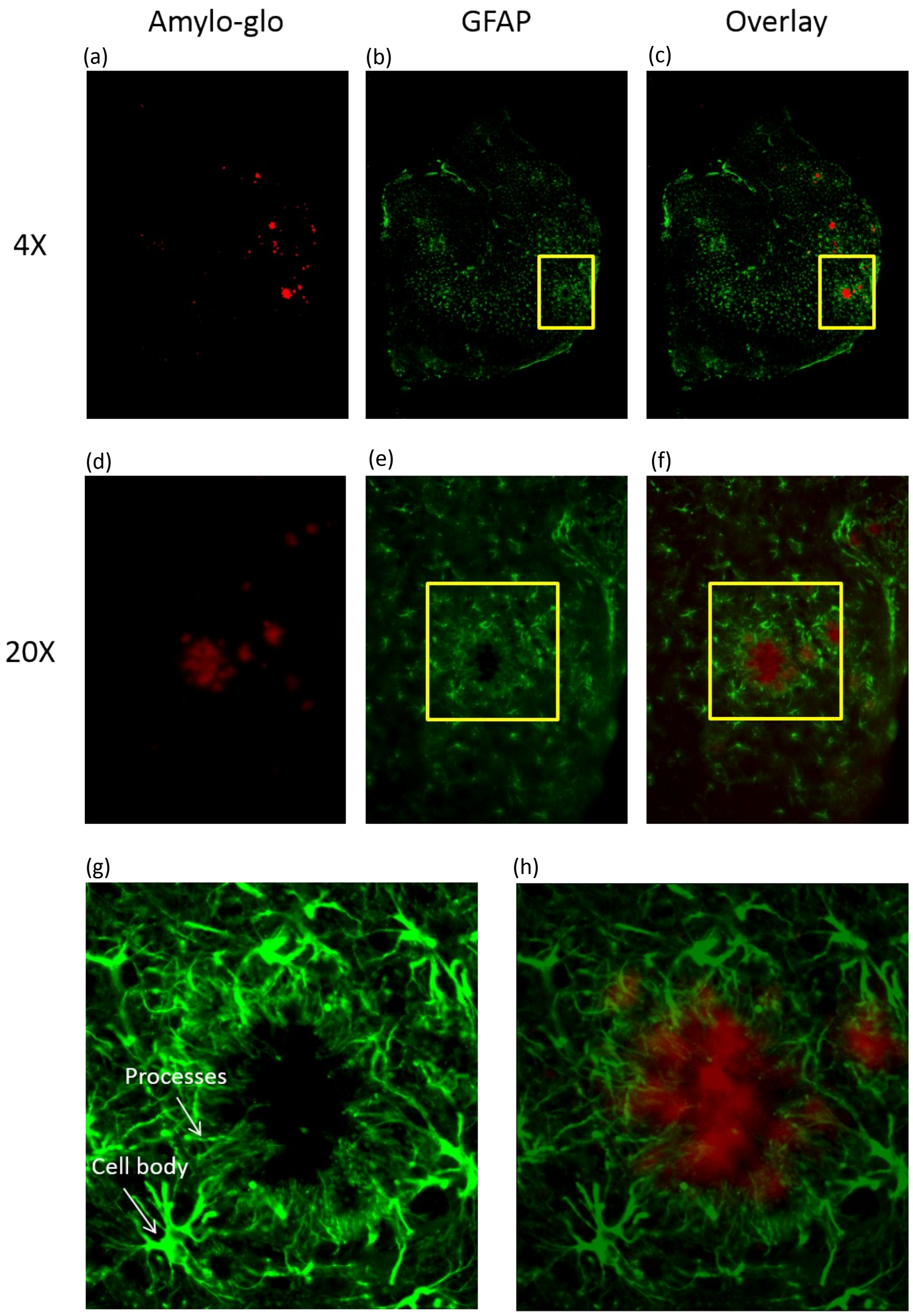

Figure SI-4. (Top panel) Immunofluorescence images at 4× magnification of a section of TG mice brain containing the hippocampus stained with **(a)** amylo-glo for Aβ peptide and **(b)** GFAP for astroglia. **(c)** Composite image formed by overlaying the fluorescence images. The box (yellow) indicates an area where fluorescence images at 20× magnification were obtained. (Middle panel) **(d–f)** Immunofluorescence images at 20× magnification of the same section. The box (yellow) defines the area of an individual large plaque. (Bottom panel) **(g,h)** Expanded views of the plaque with arrows indicating the location of processes and a cell body.

Acknowledgments

This work was generously supported by the Wellcome Trust Institutional Strategic Support Award (WT105618MA). The animals were supplied by Glaxo Smith Kline, as part of the European Union PharmacogIMI consortium. F.T. and J.H. were supported by an Alzheimer's Society fellowship.

Author contributions

F.P. and F.T. conceived, designed and supervised the project. F.T. obtained and characterized the samples. J.C.G.J., J.H., S.M., I.S., F.P. and J.N. performed the experiments. F.P., F.T. and N.S processed and analysed the data. J.T.B., A.D.R., J.H., J.N. and N.S. helped with the study design and discussion of the results. F.P. wrote the manuscript with input from all other authors.

Additional information

Competing financial interests: The authors declare no competing financial interests.

Materials & Correspondence: Correspondence and material requests should be addressed to the corresponding author: f.palombo@exeter.ac.uk.

References

1. J. Schindelin, I. Arganda-Carreras, E. Frise, V. Kaynig, M. Longair, T. Pietzsch, S. Preibisch, C. Rueden, S. Saalfeld, B. Schmid, J.-Y. Tinevez, D. J. White, V. Hartenstein, K. Eliceiri, P. Tomancak and A. Cardona, *Nat Meth*, 2012, **9**, 676-682.
2. A. C. S. Talari, Z. Movasaghi, S. Rehman and I. u. Rehman, *Applied Spectroscopy Reviews*, 2015, **50**, 46-111.
